# QED cascade with 10 PW-class lasers

**DOI:** 10.1038/s41598-017-15747-1

**Published:** 2017-11-10

**Authors:** Martin Jirka, Ondrej Klimo, Marija Vranic, Stefan Weber, Georg Korn

**Affiliations:** 1 Institute of Physics of the CAS, ELI-Beamlines Project, Na Slovance 2, Prague, 182 21 Czech Republic; 2 Faculty of Nuclear Sciences and Physical Engineering, Czech Technical University in Prague, Brehova 7, Prague, 115 19 Czech Republic; 3 GoLP/Instituto de Plasmas e Fusão Nuclear, Instituto Superior Técnico, Universidade de Lisboa, Lisbon, 1049-001 Portugal

## Abstract

The intensities of the order of 10^23–24^ W/cm^2^ are required to efficiently generate electron-positron pairs in laser-matter interaction when multiple laser beam collision is employed. To achieve such intense laser fields with the upcoming generation of 10 PW laser beams, focusing to sub-micron spot size is required. In this paper, the possibility of pair production cascade development is studied for the case of a standing wave created by two tightly focused colliding laser pulses. Even though the stronger ponderomotive force expels the seed particles from the interaction volume when a tightly focused laser beam is used, tight focusing allows to achieve cascade pair production due to the higher intensity in the focal spot. Optimizing the target density can compensate the expulsion by the ponderomotive force and lower the threshold power required for cascade pair production. This will in principle allow to produce pairs with 10 PW-class laser facilities which are now under construction and will become accessible soon.

## Introduction

With PW class laser facilities^1^ it is nowadays possible to achieve intensities of the order of 10^22^ W/cm^2^. Due to the continuing development in laser technology, 10 PW laser facilities come into operation in the next few years. These facilities^2–4^ are expected to reach intensities of the order of 10^23–24^ W/cm^2^. With such powerful laser systems, quantum electrodynamics (QED) effects start to manifest themselves in laser-matter interaction during the experiments (see ref.^5^ for an overview). In such an intense laser field, the radiation reaction force can strongly influence the interaction of a laser beam with matter since this effect reduces the energy of emitting particles and transforms their energy into gamma radiation^6–8^. Consequently, the emitted photons interacting with the strong laser field can create electron-positron pairs via the Breit-Wheeler process (BW)^9^. If the newly created BW electron or positron interacts with the strong electromagnetic (EM) field, the process of photon emission and pair creation can repeat itself, and thus a cascade pair production can develop. The increase in the number of generated pairs can lead to the depletion of the laser pulse^10,11^.

Electron-positron pairs can be generated in a standing wave formed by two colliding laser pulses when a seeding particle is placed into the interaction region^12^. The efficiency of pair production depends not only on the laser intensity, but also on polarization and on the cascade seeding. It has been found that linear polarization is more efficient in the case of non-ideal seeding of the cascade, therefore we focus on linearly polarized laser beams^13–15^. This configuration has been so far studied in terms of linearly polarized plane waves or focused paraxial beams which can not be applied in the case of tight focusing (${w}_{0}\lesssim \lambda$) even if higher-order terms of the expansion parameter *θ* = *w*_0_/*z*_*R*_ = *λ*/(*πw*_0_) are employed^16^. Here, *w*_0_ stands for the focal spot radius, *λ* is the laser wavelength and *z*_*R*_ = *πw*_0_/*λ* is the Rayleigh length. The parameter *θ* closely approximates the beam diffraction angle^17^. Nevertheless, due to the necessity to achieve laser intensities of the order of 10^24^ W/cm^2^, the laser beam has to be focused to a *λ* scale spot size, i.e. beyond the validity of the paraxial approximation. It is therefore important to use an appropriate model that can accurately describe a tightly focused laser beam. The interaction under tight-focusing undergoes additional effects (i.e. development of parallel electric field, pulse shortening^17^) that are not encountered in simulations within the paraxial approximation. The focusing to a wavelength scale focal spot can be realized by the combination of an off-axis parabolic mirror and an ellipsoidal plasma mirror^18^.

It has been reported^19^ that in the interaction of tightly-focused laser beams with a low-density target, electrons are expelled from the focal volume due to the strong ponderomotive force that prevents cascade development even at very high laser intensities of the order of 10^26^ W/cm^2^. Employment of multiple colliding laser beams leads not only to lowering the required power but also ensures more efficient seeding of the QED cascade^13,20–22^. Since the effect of target density has not been taken into account in previous studies, we study the interaction of two colliding laser pulses with an electron cloud in the case of tightly-focused laser beams for a wide range of target densities and various focal spot sizes in order to find the threshold for cascade pair production in such a configuration. It is shown that in the case of properly chosen target density, tight focusing allows to achieve the cascade generation of electron-positron pairs even at 10 PW-class laser facilities. The power is similar to what was considered in ref.^22^. However, we show that a high density is required in case of a complete simulation of the interaction dynamics.

To characterize the interaction of a charged particle with strong EM fields, two invariant and dimensionless parameters are used. The EM field can be described by the normalised vector potential ${a}_{0}=e{E}_{0}/{m}_{e}{\omega }_{0}c$, where *e* is the elementary charge, *E*_0_ is the amplitude of the EM field strength, *m*_*e*_ is the mass of electron, *ω*_0_ is the laser frequency and *c* is the speed of light^23^. As soon as *a*_0_ > 1, the electron motion starts to be relativistic^24^. The interaction of a charged particle (photon) with EM field is characterized by the parameter ${\chi }_{e}={[|({F}_{\mu \nu }{p}_{\nu }{)}^{2}]}^{\mathrm{1/2}}/{m}_{e}c{E}_{S}$ (${\chi }_{\gamma }={[|({F}_{\mu \nu }\hslash {k}_{\nu }{)}^{2}|]}^{\mathrm{1/2}}/{m}_{e}c{E}_{S}$), where ${E}_{S}={m}_{e}^{2}{c}^{3}/e\hslash \simeq 1.3\times {10}^{18}{\rm{V}}{\rm{/}}{\rm{m}}$ is the Schwinger limit field, $\hslash$ is the Planck constant, and *F*_*μv*_ is the EM field tensor^23,25^. Electron and photon four-momentum are expressed as *p*_*v*_ and $\hslash {k}_{\nu }$, respectively. For ${\chi }_{e}\ll 1$, the emission of photons is modeled as a continuous process of losing energy^26^. If ${\chi }_{e}\gtrsim 1$, emitted photons carry a large fraction of the particle’s energy and the radiation reaction has to be modeled as a step-like quantum process^27^. While for ${\chi }_{\gamma }\ll 1$ the rate of pair creation is exponentially small^23^, for ${\chi }_{e},{\chi }_{\gamma } > 1$, the interaction leads to an avalanche, i.e. the exponential growth of the electron, positron and photon numbers^11^. We define a threshold for the cascade pair production when at least one electron-positron pair per seed particle is created (same as in ref.^19^).

## Results

We performed 2D simulations with the particle-in-cell code EPOCH to study the effect of tight focusing and initial target density on cascade pair production efficiency. The target consisting of an electron cloud and immobile ions is irradiated by two counter propagating laser pulses. The target is initially neutral as the immobile ions provide a background neutralizing the electron cloud.

The circular target, whose radius is equal to the focal spot radius *w*_0_, is placed in the center of the simulation box at *x* = *y* = 0 *μ*m. It is located in the common focal spot of the two colliding laser beams. They propagate along the *x*-axis while being polarized along the *y*-direction. To describe the EM field of a tightly focused, p-polarized laser pulse we used the EM field expressions published in ref.^28^ (see Methods). Both laser pulses have the wavelength *λ* = 1 *μ*m and a Gaussian temporal profile. The full width at half maximum (FWHM) duration is 30fs. Laser beams were focused to a focal spot of radius *w*_0_ = 0.5 *μ*m − 2 *μ*m. We study pair production for a wide range of initial target density *n*_*e*_ going from 0.01*n*_*c*_ to 500*n*_*c*_, where ${n}_{c}={m}_{e}{\varepsilon }_{0}{\omega }_{0}^{2}/{e}^{2}$ is the critical electron density and *ε*_0_ is the vacuum permitivity.

As the laser pulses collide, they form a standing wave. The development of a cascade pair production strongly depends on efficient seeding, thus for seed particles it is crucial to stay in the focal spot region (i.e. within the distance of *w*_0_ from the origin) till the highest intensity of the standing wave is established. However, as the laser pulse is focused more tightly, the ponderomotive force becomes stronger and seed particles are expelled more rapidly, so the focusing acts against an efficient cascade seeding.

Nevertheless, this unwanted effect can be overcome by increasing the target density. There exists an optimal target density at which radiation pressure due to the laser photons is compensated by the electrostatic field, so that seed particles are not expelled and remain in the high-field region, and thus pair production efficiency is the highest. We can estimate the optimal target density for a simplified case assuming that seed particles are located in a circular target having the radius equal to the focal spot radius *w*_0_, and laser pulses propagate along the *x*-axis while the ponderomotive force expels seed particles in the *y*-direction. The radiation pressure along the *y*-axis, which is caused by the laser beam, can be expressed as ${P}_{rad}=\mathrm{(1}+R-T){\varepsilon }_{0}{E}_{x}{B}_{z}c/2$, where *R* and *T* stand for reflectivity and transmissivity, respectively. Let us assume that electrons are pushed away along the *y*-axis to the distance of *L* and create the electrostatic field ${E}_{es}=e{n}_{e}L/{\varepsilon }_{0}$. The distance *L* is chosen as *L* = *w*_0_/2 since at this point the radiation pressure along the *y*-direction is maximal. Assuming that the radiation pressure *P*_*rad*_ is compensated by the pressure of the electrostatic field ${P}_{es}={\varepsilon }_{0}{E}_{es}^{2}/2$ at the distance *L*, we obtain an estimate of the optimal target density as

1 $${n}_{e}^{opt}=\sqrt{\frac{8\sqrt{2}{I}_{t}{\varepsilon }_{0}\mathrm{(1}+R-T)}{\pi exp\mathrm{(1)}{w}_{0}^{3}{k}_{0}c{e}^{2}}},$$

where *I*_*t*_ is the total intensity and *k*_0_ = 2*π*/*λ*. It gives the value ${n}_{e}^{opt}=112{n}_{c}$ for the interaction of two 5 PW beams (*a*_0_ = 963) focused to *w*_0_ = 0.5 *μ*m assuming the ideal case when no laser energy is reflected. The coefficient *T* has been set as a mean value of transmission obtained from simulations for ${n}_{e}=0.01{n}_{c}-500{n}_{c}$. The calculated optimal target density ${n}_{e}^{opt}$ is in a good agreement with the value of 100*n*_*c*_ which was obtained from the simulation.

The pair production efficiency is also affected by the relativistic critical plasma density ${n}_{c\gamma }=\gamma {n}_{c}$ during the simulation^10,29^. Above the threshold for cascade pair production the target becomes opaque for the incoming laser pulses if the density of the seed and the newborn particles is too high. Since two linearly polarized laser beams are used, the relativistic critical plasma density scales as ${n}_{c\gamma }\sim {\mathrm{(1}+{a}_{0}^{2})}^{\mathrm{1/2}}{n}_{c}$. In the collision of two 5 PW beams focused to *w*_0_ = 0.5 *μ*m, each beam has the intensity $I=1.27\times {\mathrm{\ 10}}^{24}\,{\rm{W}}/{{\rm{cm}}}^{2}$ (*a*_0_ = 963) in the focal spot. The relativistic critical plasma density is achieved when the initial target density is 125*n*_*c*_. Therefore, the standing wave can not develop for targets having higher initial density. The laser intensity is always defined as the intensity in the focal spot.

**Figure 1:**
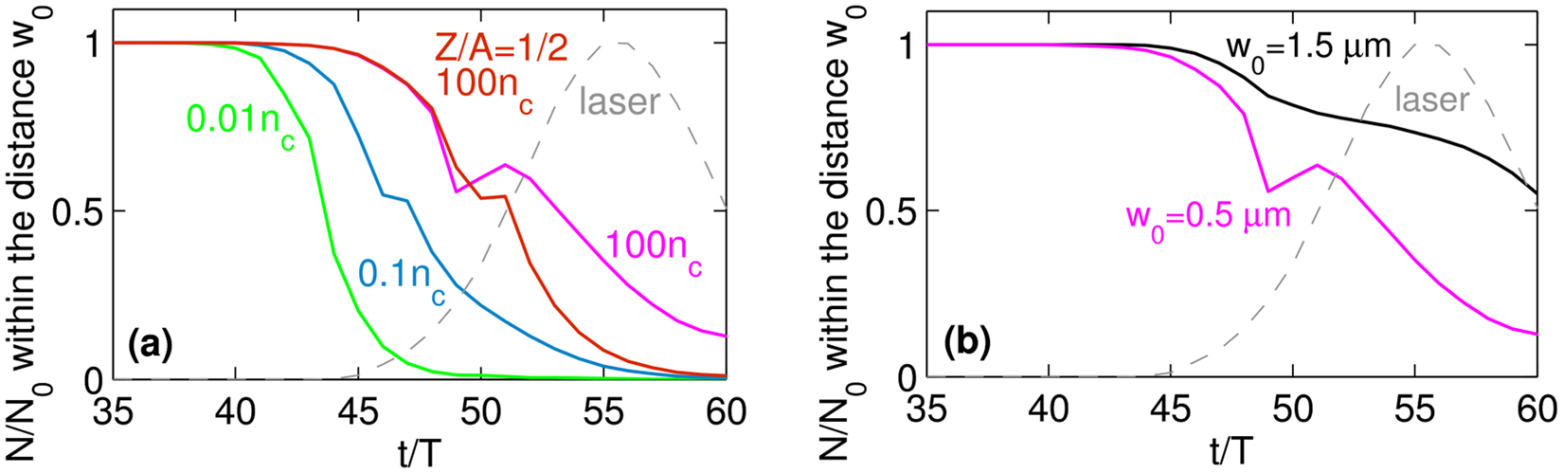
Number of seed particles *N* within the distance *w*_0_ from *x* = *y* = 0. (**a**) The effect of initial target density and ions contained in the target for *w*_0_ = 0.5 *μ*m. (**b**) The effect of focal spot radius *w*_0_ for *n*_*e*_ = 100*n*_*c*_. Gray line represents the amplitude of the standing wave. Data are calculated for threshold intensities: 1.0 × 10^24^ W/cm^2^ for *w*_0_ = 0.5 *μ*m (*a*_0_ = 854) and 6.3 × 10^23^W/cm^2^ for *w*_0_ = 1.5 *μ*m (*a*_0_ = 678). The number of particles *N* is normalized to the total sum of seed particles *N*_0_, time *t* is expressed in units of the laser period *T*.

The effect of target density on expelling the seed particles from focal spot region is illustrated in Fig. 1a for the case of *w*_0_ = 0.5 *μ*m and *I* = 1 × 10^24^ W/cm^2^ (*a*_0_ = 854). It shows the number of seed electrons *N* within the distance of *w*_0_ from the origin for the case of three different target densities (0.01*n*_*c*_, 0.1*n*_*c*_ and 100*n*_*c*_) when immobile ions are used. It also shows the situation for density 100*n*_*c*_ and ions with Z/A = 1/2 where *Z* is the atomic number and *A* is the mass number. This will be discussed in the next section.

When the target density is very low, seed particles escape the focal spot region even before the laser pulses arrive. That prevents the cascade developing even at very high laser intensities^19^. As we increase the target density (compare cases for 0.01*n*_*c*_ and 0.1*n*_*c*_), more seed particles experience the field of the formed standing wave. When the optimal target density is used (100*n*_*c*_), a significant fraction of seed particles stay in the focal spot region till the main part of laser pulses comes, and, therefore, the pair production can develop. As the standing wave gets created, the target is compressed at first. Then the seed particles are rearranged into the positions of electric field nodes of the arising standing wave^15^. During this process, the seed particles can be pushed away from the focal spot region and then, as they are attracted to the electric field nodes, come back to this area. Therefore, the abrupt increase in the lines for 0.1*n*_*c*_ and 100*n*_*c*_ arises. The situation for targets of higher density will be discussed later in this section.

Figure 1b is showing the situation in the case of threshold intensities for cascade pair production at *n*_*e*_ = 100*n*_*c*_ when laser pulses are focused to *w*_0_ = 0.5 *μ*m and *w*_0_ = 1.5 *μ*m and immobile ions are used. The corresponding laser intensity for each beam are 1 × 10^24^ W/cm^2^ (*a*_0_ = 854) and 6.3 × 10^23^ W/cm^2^ (*a*_0_ = 678), respectively. Faster expulsion of seed particles from the focal spot area is observed in the case of tight focusing. Nevertheless, the higher intensity achieved with tight focusing compensates for the smaller interaction volume, and, therefore, cascade pair production can be launched by only a fraction of initial seed particles provided they pass through the strong-field region of the standing wave. As a result, seed particles achieve higher values of the *χ*_*e*_ parameter, enhance photon emission, and, consequently, pair production. Moreover, tight focusing requires less laser power to initiate the pair production provided that the target density is properly chosen.

**Figure 2:**
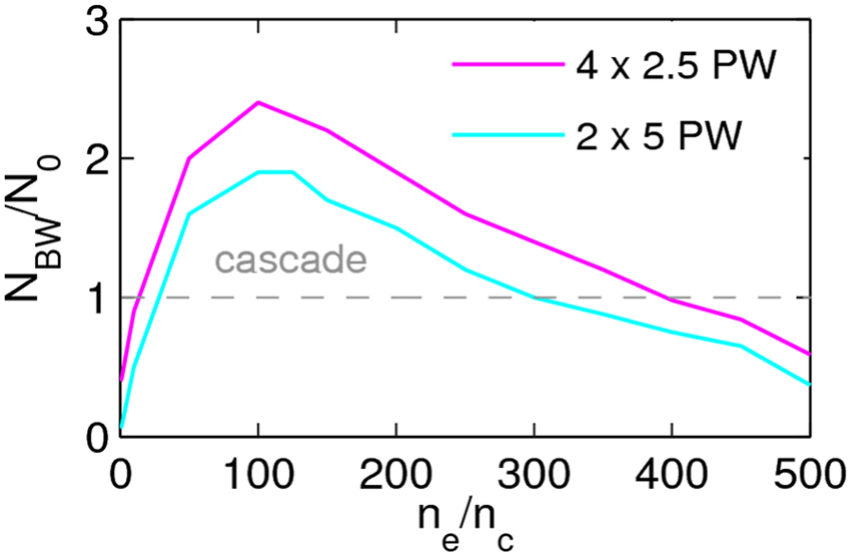
Number of BW pairs *N*_*BW*_ per one seed particle for two- (cyan) and four-beam (magenta) collision with a target of varying density. The overall laser power of 10 PW is divided into the corresponding beams focused to *w*_0_ = 0.5 *μ*m.

**Figure 3:**
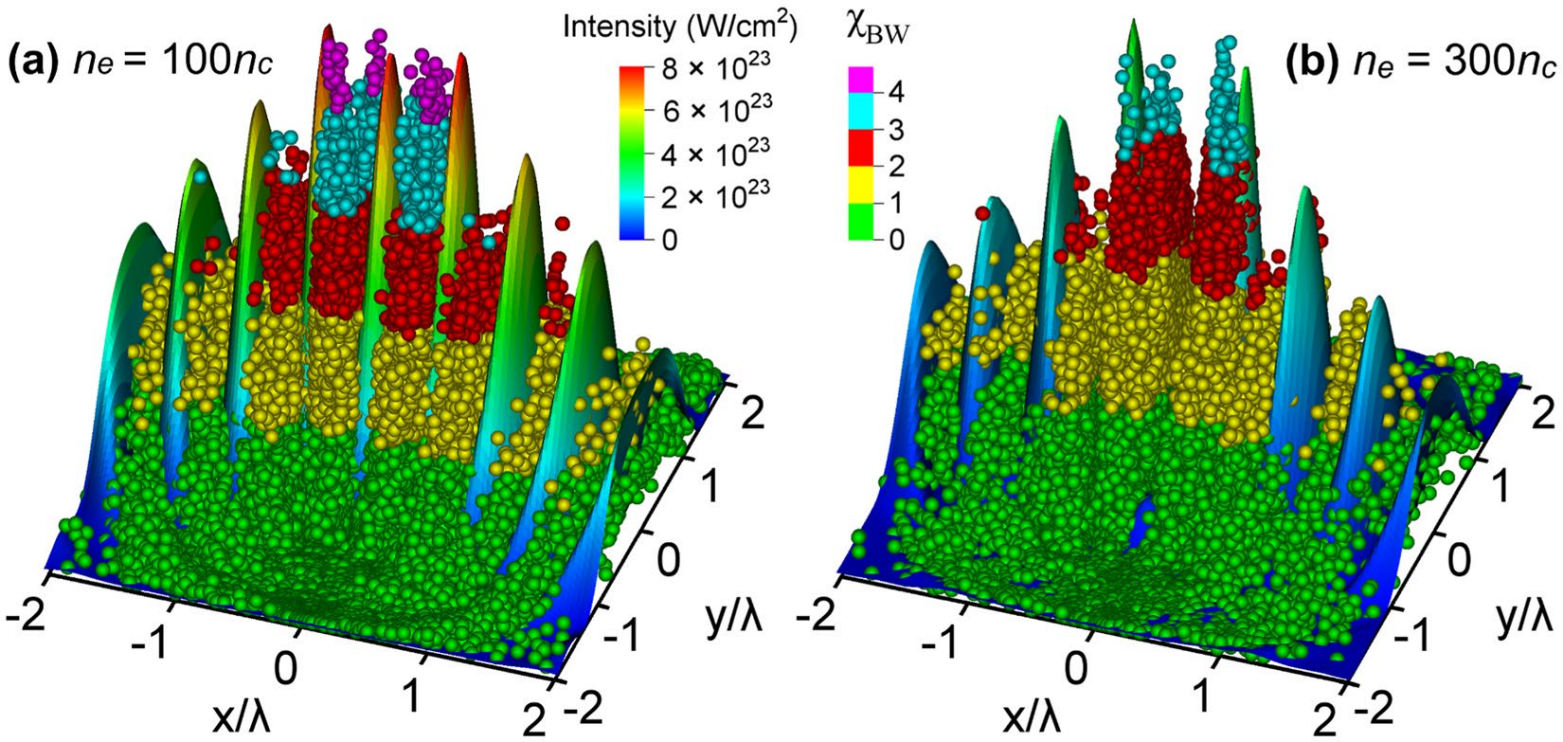
Distribution of BW electrons in the standing wave in the case of initial target density (**a**) 100*n*_*c*_ and (**b**) 300*n*_*c*_.

The ratio of newly created pairs *N*_*BW*_ to the number of seed particles *N*_0_ for different target densities is given in Fig. 2 for collision of two beams (2 × 5 PW) focused to *w*_0_ = 0.5 *μ*m. The efficiency of pair production grows with increasing target density up to the optimal target density 100*n*_*c*_ at which the ratio is the highest, 1.9, which corresponds to 1.7 × 10^17^ created BW pairs. Going to higher target densities, the pair production efficiency decreases. As reported in refs^10,29^ once a sufficient number of BW pairs are created, the relativistic critical plasma density can be reached during the interaction such that a fraction of the laser gets reflected. As a result, the standing wave is not established in the focal plane (see Fig. 3a,b). On the other hand, in the case of the optimal target density, the structure of the standing wave is not disturbed. As a consequence, particles in the vicinity of the focal spot acquire higher values of *χ*_*e,BW*_ in comparison to the case when the laser beam is reflected from the relativistic critical surface of the compressed target. Since the optimal target density allows the standing wave to fully develop, emitted photons can experience the stronger EM field that leads to higher values of the *χ*_*γ*_ parameter. This results in a more efficient pair production.

## Discussion

**Figure 4:**
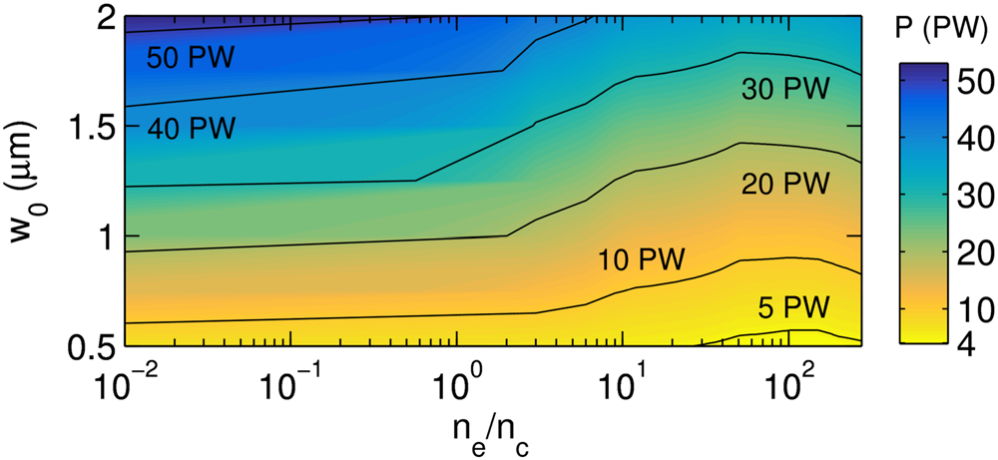
The minimal required laser power *P* per beam that enables to achieve the threshold of cascade pair production for the given focal spot radius *w*_0_ and target density *n*_*e*_.

The results of our simulations are summarized in Fig. 4, showing the required laser power threshold *P* per one beam for cascade pair production in the case of *w*_0_ = 0.5 *μ*m–2 *μ*m and target densities *n*_*e*_ = 0.01*n*_*c*_–200*n*_*c*_. For a fixed beam power, there exists a set of values *w*_0_ and *n*_*e*_/*n*_*c*_, at which the threshold for cascade pair production can be achieved. From the simulations follows that upcoming 10 PW (2 × 5 PW) laser facilities will require tight focusing of short laser pulses below *w*_0_ = 0.6 *μ*m to initiate cascade pair production while the initial target density should be approximately 100*n*_*c*_. For example, in the case of *w*_0_ = 0.5 *μ*m, two 4 PW laser beams are needed to launch the cascade pair production. Both, the tight focusing and high-density target have to be used in this case. By contrast, this is not required in the case of a more powerful laser system. When several tens of PW laser power are available, the cascade pair production can be achieved for tight focusing and a low-density target as well as for the case when both, target density and the focal spot radius, are increased. By increasing the target density, the requirements for the cascade development can be reduced by 50% for a given focal spot radius *w*_0_. As shown in Table 1, as the spot size grows, more laser power is required for cascade pair production despite the fact that the threshold intensity declines. Using longer laser pulses should lead to a more efficient pair production, however the effect of laser field depletion and reflection become more significant since a high-density electron-positron plasma would be created. Nevertheless, the threshold power and intensity would be reduced as the cascade will have more time to develop.

**Table 1:** Threshold intensity *I*, power *P* and the normalised vector potential *a*_0_ of one beam focused to *w*_0_ and the corresponding initial target density *n*_*e*_ required to launch the cascade pair production. The wavelength of the laser beam is *λ* = 1 *μ*m.

*w*_0_(*μ*m)	*a*_0_	P(PW)	*I *(W/cm^2^)	*n*_*e*_/*n*_*c*_
0.5	854	3.9	1.0 × 10^24^	100
1.0	730	11.5	7.3 × 10^23^	75
1.5	667	21.5	6.1 × 10^23^	50
2.0	634	34.5	5.5 × 10^23^	40

In our simulations we assumed perfect timing and alignment of the colliding laser pulses. To assess the influence of non-ideal configuration of the interaction, we performed simulations for the case when two 5 PW laser pulses (*a*_0_ = 963, *w*_0_ = 0.5 *μ*m) interact with a target of density 100*n*_*c*_ while the laser pulses were not perfectly aligned. We induced a shift *l* of the second laser beam axis along the *y*-direction. The values of pair production efficiency for these non-ideal cases are shown in Table 2. We also assess the role of phase synchronization in perfectly aligned laser collision. In the case when the laser pulses have the opposite phase, the pair production efficiency drops only by 9%. Despite the fact that amplitude of the standing wave is not established in the center of simulation box in this case, particles are pushed by the ponderomotive force to the position of electric field nodes where they emit. Therefore, the alignment of collision has a higher impact on pair production efficiency than the phase synchronization of the colliding laser pulses. As is shown in Table 2, pair production efficiency drops by almost 50% when the displacement of colliding laser beams by 2*w*_0_/3 is induced along the *y*-direction.

**Table 2:** Number of newborn electron-positron pairs per one seed particle for the collision of two 5 PW laser pulses (*a*_0_ = 963, *w*_0_ = 0.5 *μ*m) with a target of density 100*n*_*c*_ when the laser axis of the second beam is shifted by a distance of *l* in the *y*-direction.

*l*	*N*_*BW*_/*N*_0_
0	1.94
*w*_0_/3	1.09
2*w*_0_/3	0.95
*w*_0_	0.06

We compare pair production efficiency in the case where 10 PW laser beam is divided into two (2 × 5PW, *a* = 963) or four (4 × 2.5 PW, *a*_0_ = 684) tightly-focused (*w*_0_ = 0.5 *μ*m) colliding laser beams. Polarization and laser pulse duration remain the same as in the previous case. In the four-beam collision, the target is symmetrically irradiated by laser pulses propagating along the *x* and *y* axes, thus, the two-dimensional standing wave is set up^13,22,30^. We found that there is no big difference in the number of generated electron-positron pairs in these two interactions for any of the initial target densities (see Fig. 2). Therefore, in contrast to the focusing within paraxial approximation, the benefit of more robust seeding in the collision of four tightly-focused laser beams is not evident. For comparison, the threshold of the cascade pair production requires 10 PW for each of four laser beams when *w*_0_ = 1.5 *μm*. This is equal to the overall laser power needed for achieving pair production in the case of two or four colliding laser beams when *w*_0_ = 0.5 *μ*m. This favours tight focusing regardless of the number of colliding laser beams since it requires less power and allows development of cascade pair production even for a two-beam setup.

To assess the role of ions on pair production efficiency, we performed simulations where two laser beams (2 × 5 PW, *a*_0_ = 963, *w*_0_ = 0.5 *μ*m) interact with a target of density 100*n*_*c*_ composed of electrons and mobile ions with *Z*/*A* = 1/2. As shown in Fig. 1a, three times lower number of seed particles can experience the amplitude of the formed standing wave in comparison with the case of immobile ions when the same target density is used. This is due to the fact that the whole target expands and the seed particles are not confined to the very small interaction volume so efficiently. The resulting number of created BW pairs is then three times lower. Nevertheless, when a different shape of target is used, we can obtain the similar number of pairs as in the case of circular target with immobile ions. For example, if laser pulses are incident on a 1 *μ*m thick flat foil target that includes electrons and ions with *Z*/*A* = 1/2, the absolute number of generated pairs is only by 2% lower than in case of circular target with immobile ions while both targets are having the optimal density.

**Figure 5:**
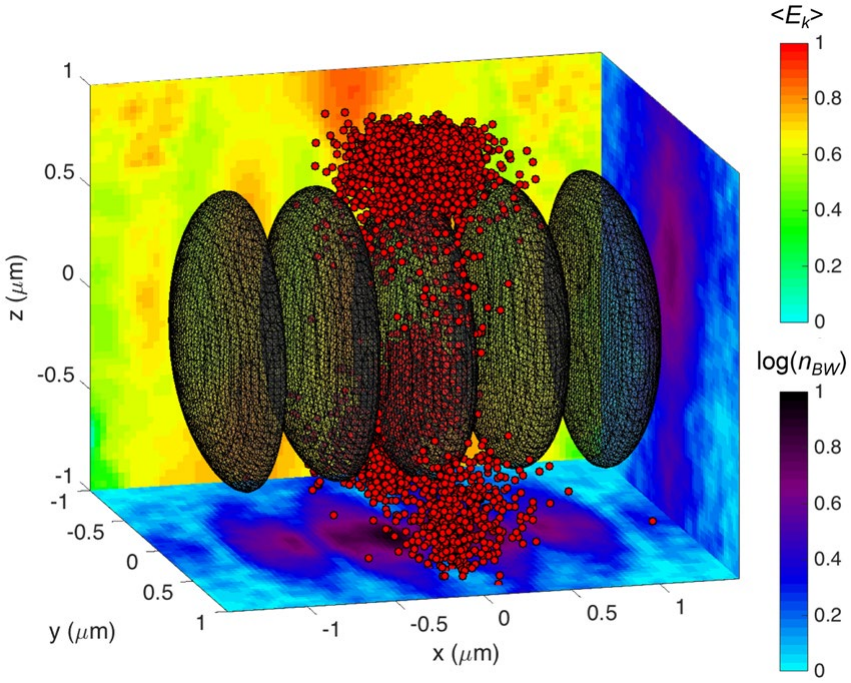
Laser-target interaction in 3D for *w*_0_ = 0.5 *μ*m and *a*_0_ = 1200. Iso-surfaces correspond to laser intensity 5 × 10^23^ W/cm^2^, red bullets to BW positrons having the relativistic factor *γ* > 1500. Mean kinetic energy $\langle {E}_{k}\rangle$ of BW positrons at *y* = 0 *μ*m is projected onto the (*x*, *z*) plane. Density of BW positrons *n*_*BW*_ at *z* = 0 *μ*m and *x* = 0.25 *μ*m is projected onto the (*x*, *y*) and (*y*, *z*) plane, respectively. All projected quantities are normalized to the maximum values and the density is in the log scale.

In order to check the validity of our results and get more realistic insight into the interaction of two linearly polarized laser pulses which are tightly focused, we carried out additional 3D simulations. The 3D case takes into account ejection of the seed particles perpendicular to the direction of laser pulse polarization, which is caused by the ponderomotive force and can not be accounted for in the 2D case. This effect results in a lower efficiency of pair production since fewer particles can experience the strong EM field of the established standing wave, and, therefore, more laser power is required to achieve the pair production threshold. According to the 3D simulation, when laser pulses interact with a spherical target having the density 100*n*_*c*_ and the radius equal to *w*_0_ = 0.5 *μ*m, 8 PW per each laser beam is necessary. Figure 5 is showing the density and mean kinetic energy of BW positrons in this interaction. BW positrons are trapped at electric field nodes, while the ones, that are expelled from the focal spot region, acquire the highest energy.

However, the required laser power threshold might be lowered. First, the target shape should be optimized so that more seed particles could remain in the interaction region till the standing wave is established. Second, laser pulses of longer duration could be used for the interaction as it would lead to a more efficient pair production^29^. We have performed the study for short 30 fs laser pulses, but the new 10 PW laser facility ELI-Beamlines^2^ will provide laser pulses of duration 150 fs. The longer duration of the standing wave will result in higher number of created pairs. That would lead to further lowering the threshold for cascade pair production. To asses these effects, a more detailed study involving 3D simulations is necessary. Furthermore, our additional 3D simulations verified that the threshold power for cascade pair production is the lowest with the optimal target density when tight focusing is employed, which is in agreement with our model and 2D simulations.

Tight focusing could be a key to an efficient pair production at 10 PW-class laser facilities in the setup where two laser beams interact with a target of a properly chosen density. We have shown that using a target with an appropriate density can help balance the effect of expelling seed particles from the high intensity region. Less laser power is required to initiate the cascade when tight focusing is employed, which is favourable for experiments at the upcoming multi-PW laser facilities. Moreover, achieving the high-intensity field using the tight focusing leads to a more efficient two-beam collision in comparison to previous studies. Finally, our results do not show an advantage of a more efficient seeding using a four-beam collision for this setup.

## Methods

### Numerical Modelling

It has been shown that in the case of colliding laser beams, the highest efficiency of pair production is achieved when p-polarization is used^13,14^. We analyze the laser-target interaction with p-polarized laser pulses using 2D and 3D particle-in-cell simulations with the code EPOCH in which the stochastic nature of radiation emission is modeled using Monte Carlo technique. The electron-positron pair creation via the Breit-Wheeler processes is also implemented in the code. For more details about the code please see the ref.^31^.

In our 2D simulations, the dimensions of the simulation box are 40 *μ*m × 100 *μ*m, the spatial resolution is *λ*/30 in both directions and we used 100 particles-per-cell. Convergence tests were performed to verify the simulation resolution used in the paper was sufficient for studying the threshold of the cascade pair production. To obtain the data which is summarized in Fig. 4, we performed for each value of the focal spot radius (*w*_0_ = 0.5 *μ*m, 1.0 *μ*m, 1.5 *μ*m, 2.0 *μ*m) and for each value of the target density (*n*_*e*_ = 0.001*n*_*c*_, 0.01*n*_*c*_, 0.1*n*_*c*_, 1*n*_*c*_, 10*n*_*c*_, 50*n*_*c*_, 100*n*_*c*_, 110*n*_*c*_, 125*n*_*c*_, 150*n*_*c*_, 200*n*_*c*_, 250*n*_*c*_, 300*n*_*c*_) a series of 2D simulations to find the minimal power required for achieving the threshold for cascade pair production. The remaining data points are obtained by linear interpolation in Fig. 4. The data presented in Table 1 are obtained from the simulations. In the 3D case, the simulation box has dimensions 7 *μ*m×7 *μ*m×7 *μ*m. The other parameters of the simulations remain the same as in the 2D case.

To model the EM field of a tightly focused laser beam we used the description which is valid within the constrained space-time region given by the conditions $x\lesssim {z}_{R}$ and $c\tau \lesssim {z}_{R}$, where *τ* is the pulse duration^28^. We calculated the EM field at the focal plane (*x* = 0 *μ*m) for the whole duration of the laser pulse. By propagating the fields using finite-difference time-domain (FDTD) method, we obtained the description of EM fields at any plane parallel to the focal one for all time steps. These fields are then used as an input describing tightly-focused laser beams at the boundaries of the simulation box with the direction of their propagation reversed. Simulating tightly focused beams can be a challenge for FDTD algorithm, because of possible numerical errors. However, we have verified against the analytical description from eq. (28) in ref.^28^ that the cumulative error due to the propagation of the electromagnetic fields through FDTD remains below 1% of the field amplitude in the laser focus.

### Data Availability Statement

The datasets generated and analyzed during the current study are available from the corresponding author on request.
